# Bipolar configuration with twisted loop defect in chiral nematic droplets under homeotropic surface anchoring

**DOI:** 10.1038/s41598-017-15049-6

**Published:** 2017-11-06

**Authors:** Mikhail N. Krakhalev, Anna P. Gardymova, Oxana O. Prishchepa, Vladimir Yu. Rudyak, Alexander V. Emelyanenko, Jui-Hsiang Liu, Victor Ya. Zyryanov

**Affiliations:** 1 Kirensky Institute of Physics, Federal Research Center – Krasnoyarsk Scientific Center, Siberian Branch, Russian Academy of Sciences, Krasnoyarsk, 660036 Russia; 2 Institute of Engineering Physics and Radio Electronics, Siberian Federal University, Krasnoyarsk, 660041 Russia; 3 Faculty of Physics, Lomonosov Moscow State University, Moscow, 119991 Russia; 4 Department of Chemical Engineering, National Cheng Kung University, 70101 Tainan, Taiwan

## Abstract

Optical textures and appropriate orientational structures have been studied within droplets of chiral nematic dispersed in polymer assigning the homeotropic anchoring. The helix axis of the chiral structure inside droplets forms the bipolar configuration. The optical droplet textures were analysed in the unpolarised light, analyser switching-off scheme and in crossed polarisers. The twisted loop defect reveals itself convincingly in all schemes. Its appearance at the optical patterns of the chiral nematic droplets has been examined depending on their size and the aspect direction. The existence of the defect has been verified by the structural and optical calculations. The effect of an electric field on both the defect line shape and the orientational structure of chiral nematic has been studied.

## Introduction

Chiral nematic liquid crystals (CLC) are characterized by the twisted structure of the director field. Interaction of CLC with its envelope can disturb the twisted structure uniformity and cause a variety of director configurations. The latter depend on the boundary conditions (a preferable director orientation at the interface, surface anchoring strength) and the CLC material parameters (elastic modulus, intrinsic helix pitch). For example, the destruction of the uniform helical structure followed by the formation of a number of stable and meta-stable topological defects was observed within the CLC layer with homeotropic anchoring^1–8^. The complex 3D systems of linear defects (twisted loops, lines-links and knots) are formed in CLC containing the colloidal microspheres^9–13^. The defect topology in these systems also depends on the number and size of spherical particles. For instance, a twisted loop is shaped around a single particle assigning the homeotropic boundary conditions for CLC. In this case, the number of the loop turns depends on the ratio of helix pitch and particle size^13^.

CLC droplets possess a variety of orientation structures so that different topological defects are possible due to their confined envelope. Under the tangential anchoring, the stable twisted bipolar structure^14–17^ and the structures with diametrical χ^+1^ or radial χ^+2^ dislocations^16–20^ can be realized. A series of meta-stable structures has been predicted by the simulation method^21^. The uniform helix axis distribution^18,22,23^ and the double twisted structure^23^ can be observed within the droplets with a weak anchoring at the nematic-isotropic interface in CLC.

CLC droplets with the homeotropic boundary conditions have been less examined. The structures with the point defect in the bulk of CLC^24^ or at the surface^17^ under conditions *p*_0_/*d* ≫ 1, the structures with the bipolar distribution of CLC axes^18,25^ or with the radial χ^+2^ dislocation^24^ for *p*_0_/*d* ≪ 1 can arise depending on the *p*_0_/*d* ratio of intrinsic CLC helix pitch *p*_0_ to the droplet size *d*. The structure with the equatorial disclination was observed in the case of *p*_0_/*d* ≪ 1 and *p*_0_/*d* ~ 1^17^. The formation of a series of singular nematic disclination lines with winding number – 1/2, knots and links inside the droplets depending on the relative chirality parameter *N*_0_ = 2*d*/*p*_0_ was theoretically evidenced^26^. The layer-like structure, locally similar to the CLC ground state, with the twisted disclination loop disposed near the interface appears within the droplets for *N*_0_ > 5. The patterns of CLC droplets and their transformations were examined in ref.^27^. However, their possible orientational structures were not analysed in detail. By the methods of polarising optical and scanning confocal microscopy, the defects with winding number +1 were shown to appear inside the droplets with ratio *p*_0_/*d*~1^28^. Three-dimensional topology of the director distribution at a different *N*_0_ was analysed by the method of fluorescent confocal polarising microscopy^29,30^. The structures with odd numbers of the point topological +1 defects have been shown to realize within the droplets. The positions of point defects in the bulk were stabilized by the twisted structures resembling the skyrmions and torons.

In this paper we present the results of the experimental study of CLC droplets in the polymer dispersed chiral-nematic liquid crystals (PDCLC) films with homeotropic anchoring and analyse in detail orientation structures of the droplets. A variety of director configurations is observed within the samples under study. We focused our attention only on the CLC droplets with twisted defect loop in order to demonstrate obviously the defect patterns depending on the chirality *N*, bipolar axis orientation relatively to the aspect direction and applied voltage.

## Results and Discussion

### Optical textures and orientation structures of CLC droplets

**Figure 1:**
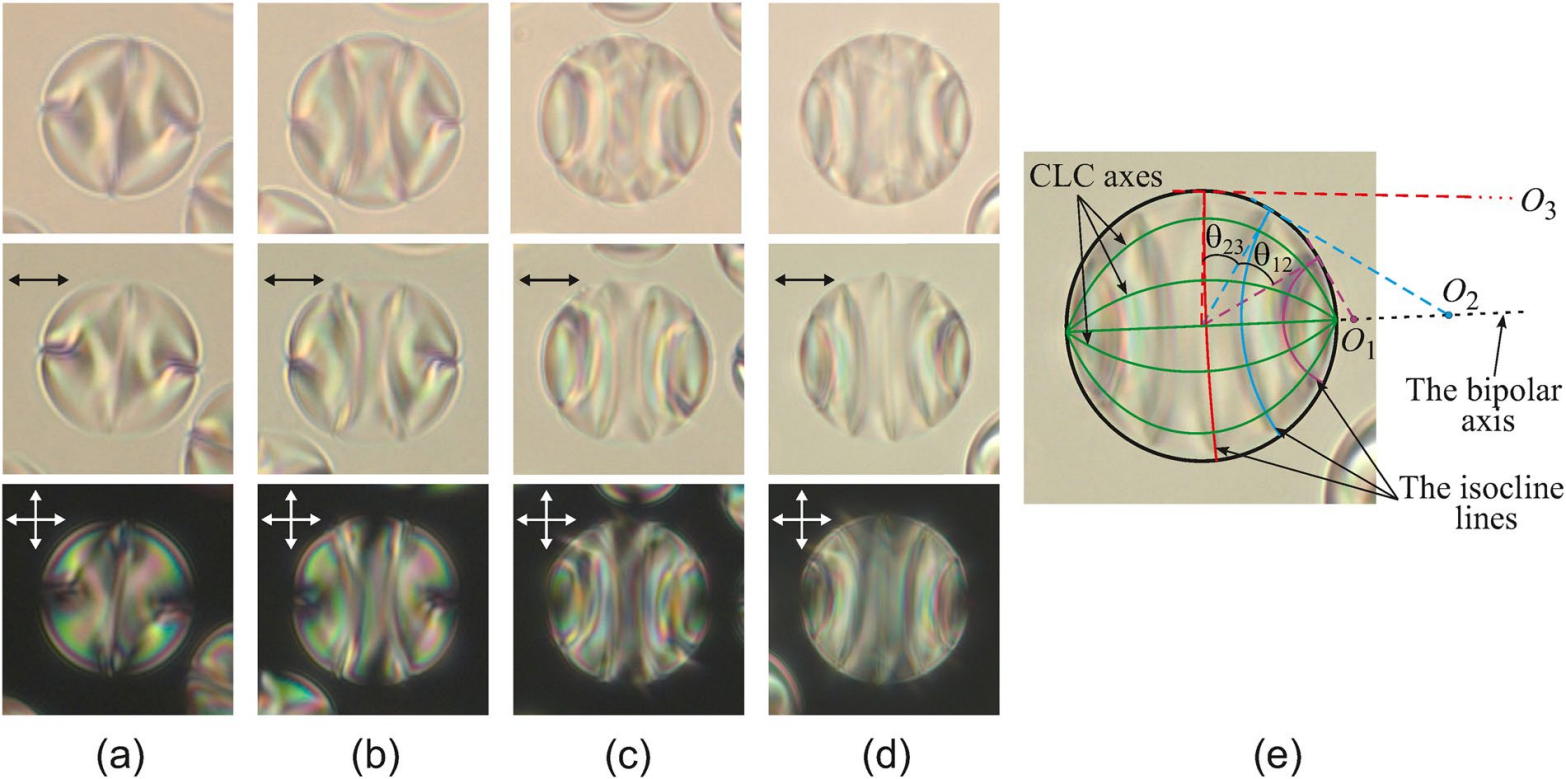
Micro-photos of CLC droplets in the unpolarised light (top row), in polarised light (middle row) and in crossed polarisers (bottom row). Droplet’s sizes are 14 μm (**a**), 17 μm (**b**), 19 μm (**c**) and 21 μm (**d**). Micro-photo of the droplet (**d**) presented in the middle row with the scheme of the isoclinal lines, CLC’s axes and bipolar axis (**e**). *O*_1_, *O*_2_, *O*_3_ are the centers of circular arcs of the isoclinal lines, θ_12_ ≅ θ_23_ are angular distances between the isoclinal lines on the droplet’s border. The polariser’s directions are indicated by the double arrows here and below.

PiBMA-polymer assigns the homeotropic anchoring for the E7 nematic^31^. In the sample under study the structure with the point defect-hedgehog in the droplet’s centre, typical for the homeotropic anchoring, is formed inside the CLC droplets of 5 μm size. It means that the polymer PiBMA also specifies the homeotropic anchoring for the CLC mixture studied. At that, a rich variety of optical textures of CLC droplets is observed (see Supplementary Figure 1). A distinctive structure revealed as the curved lines is observed within droplets of 14–30 μm size in the film plane (Fig. 1). The optical texture specific to a structure with the bipolar distribution of CLC axes is observed in the direction perpendicular to the bipolar axis^18,25^. CLC axes converge in two antipodal points (the poles of the bipolar structure). The shape of the observed curved lines is close to the circular arcs whose centers are along the bipolar axis. Here these lines cross perpendicularly the droplet’s border, and the angular distances θ_12_ and θ_23_ (Fig. 1e) between the adjacent areas are approximately equal. The curved lines observed inside CLC droplets are areas at the microscope focal plane where the director is oriented at the same angle (parallel or perpendicular) to the film plane. Therefore, these lines can be named isoclinal by analogy with ref.^32^.

### Twisted loop defect

A distance between the isoclinal lines corresponds to the half of the CLC’s helix pitch. It varies from a maximal value near the droplet border to a minimal one in its centre. The parameter *N*, which is the real number of π-turns of the director along the bipolar axis, is convenient to analyse the texture. An analytical expression for the parameter *N* was obtained in ref.^33^:

1 $$N={N}_{0}-\frac{b}{d}=\frac{2d}{{p}_{0}}\,-\,\frac{b}{d},$$

where *d* is the droplet size, *b* is a coefficient depending on the intrinsic helix pitch *p*_0_. The CLC mixture with *b* = 46.7 μm was studied in this paper.

A sharp distortion of the director field accompanied by the formation of the linear defect near the surface must take place in the structure with the bipolar distribution of CLC axes, which is confirmed by the results of computer simulation of the droplet structure with homeotropic anchoring at *N*_0_ > 5^26^. In this case, the layer-like structure with the double helix (twisted loop) defect is formed near the surface. The linear surface ring defect in the achiral nematics can be observed under the optical microscope if the defect plane is perpendicular^34^ or at some nonzero angles to the film plane^35^.

**Figure 2:**
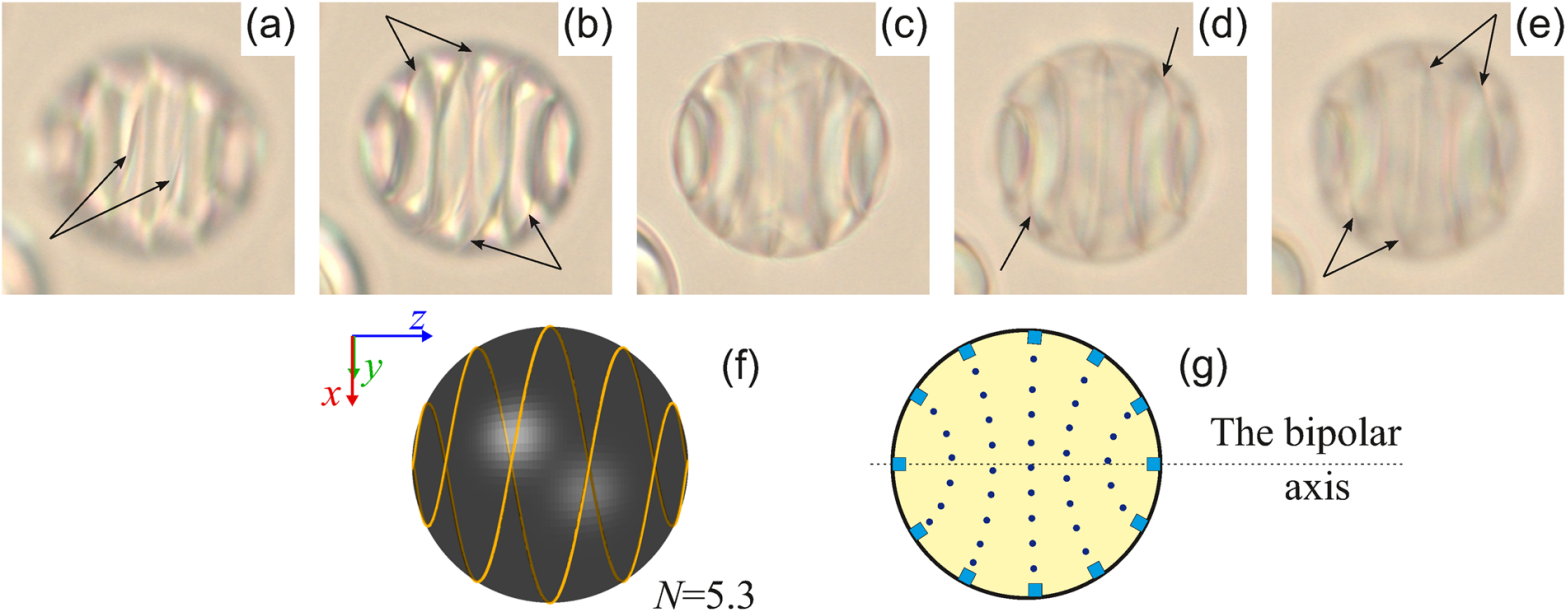
Micro-photos of the droplet shown in Fig. 1d (top row) for various focusing of microscope. The focal point is located approximately at 1/6 (**a**), 2/6 (**b**), 3/6 (**c**), 4/6, (**d**), 5/6 (**e**) of the lateral size of droplet from the top border. The linear defect like a double helix (twisted loop defect) is indicated by the arrows here and below. The schemes of the linear defect *L* at *N* = 5.3 (**f**) and isoclinal (dotted) lines in the cross-section where the director is oriented perpendicularly to the sectional plane (**g**). The sections of linear defect are indicated by the blue squares.

Moving the focus of microscope objective, a rather sharp picture of the linear defect can be resolved (Fig. 2). At that, a series of lines connecting the opposite parts of the isoclinal lines near the visible droplet border is clearly seen (Fig. 2a–e). The same pattern can result from the double left-handed helix defect. In this case, the linear defect must be near the surface area where the director orientation assigned by the twisted structure in the bulk tends to be strictly parallel to the border. The fact that the linear defect at the visible droplet border coincides with the isoclinal line suggests that these lines correspond to the director orientation perpendicular to the film plane.

Figure 2f demonstrates the scheme of the double left-handed helix *L* belonging to the spherical surface of the unit radius whose Cartesian coordinates can be described by the following parametric equations:

2 $$\{\begin{array}{ccl}x & = & {(-1)}^{k+1}\,\sin \phi \cdot \,\sin (N\phi )\\ y & = & {(-1)}^{k}\,\sin \phi \cdot \,\cos (N\phi )\\ z & = & \,\cos \,\phi \\ 0 & \le  & \phi \le \pi ,k=1,2\end{array},$$

at *N* = 5.3 in correspondence with equation (1). Here *z*-axis coincides with the bipolar axis of CLC droplet, φ is an angle between the position vector of a definite point on the unit sphere and *z*-axis. So, the double spiral (twisted loop) is the combination of two symmetrical spirals relatively to *z*-axis. Two diametrically opposite defect points are in each *xy* cross-section of the droplet (in more detail see Supplementary Figure 2). Figure 2g shows the scheme of an appropriate director configuration in the droplet central cross-section. The director is oriented perpendicularly to the section plane at particular circular arc (dotted) lines with the centers located at the bipolar axis.

### Dependence of optical textures on the droplet size and orientation of bipolar axis

**Figure 3:**
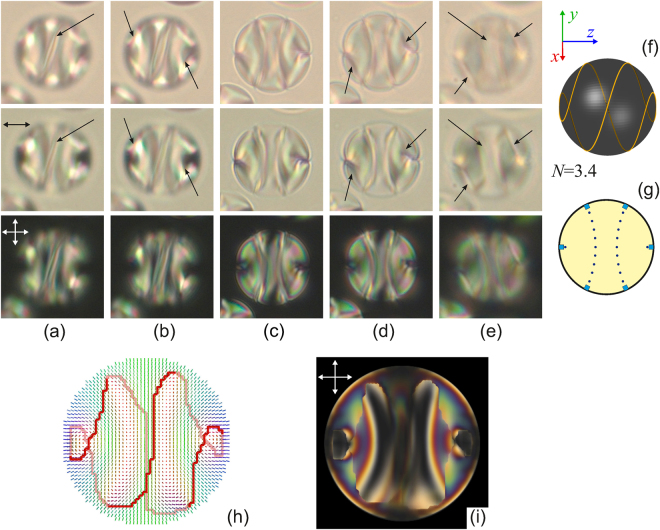
Micro-photos of the CLC droplet (Fig. 1b) made in the unpolarised light (top row), polarised light (middle row) and crossed polarisers (bottom row) for various focusing of microscope. The focal point is located approximately at 1/6 (**a**), 2/6 (**b**), 3/6 (**c**), 4/6, (**d**), 5/6 (**e**) of the lateral size of droplet from the top border. The scheme of the linear defect *L* at *N* = 3.4 (**f**) and the isoclinal lines in the cross-section where the director is oriented perpendicularly to the sectional plane (**g**). The calculated droplet structure in cross-section (**h**). The director vectors are colored in correspondence with the direction (green along y-axis, red along x-axis, and blue along z-axis). Red solid thick and opaque thick lines show linear disclination in near and far half-spaces, correspondingly. The simulated droplet texture in crossed polarisers (**i**).

**Figure 4:**
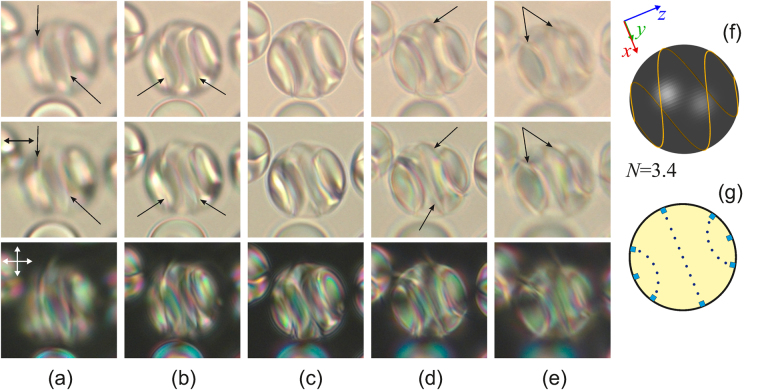
Micro-photos of the CLC droplet made in the unpolarised light (top row), polarised light (middle row) and crossed polarisers (bottom row) for various focusing of microscope. The focal point counts from the top border and is located approximately at 1/6 (**a**), 2/6 (**b**), 3/6 (**c**), 4/6, (**d**), 5/6 (**e**) of the lateral size of droplet. The scheme of the linear defect *L* at *N* = 3.4 (**f**) and the isoclinal lines in the cross-section where the director is oriented perpendicularly to the sectional plane (**g**). The droplet size is 17 μm.

**Figure 5:**
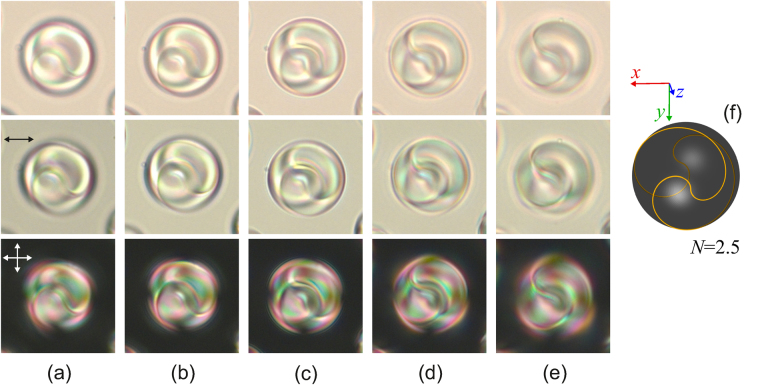
Micro-photos of the CLC droplet made in the unpolarised light (top row), polarised light (middle row) and crossed polarisers (bottom row) for various focusing of microscope. The focal point is located approximately at 1/6 (**a**), 2/6 (**b**), 3/6 (**c**), 4/6, (**d**), 5/6 (**e**) of the lateral size of droplet from the top border. The scheme of the linear defect *L* for *N* = 2.5 (**f**) when the bipolar axis (*z-*axis) is oriented at the 25° angle to the aspect direction. The droplet size is 17 μm.

Similar patterns of the left-handed double helix linear defects are observed inside droplets with a smaller *N* number. At that, the optical texture and the double helix pattern depend on the orientation of bipolar axis with respect to the aspect direction. The droplets with *d* = 17 μm (Figs 3–5) demonstrate this feature very distinctly for a relatively small *N*.

Figure 3 shows the droplet with the bipolar distribution of CLC axes at *N* ≅ 3.4 for the case of bipolar axis aligning in the film plane. Two isoclinal lines located symmetrically relative to the droplet center are clearly seen. Various areas of the linear double helix defect can be scanned moving the microscope focus. The double helix line *L* [see equation (2)] at *N* = 3.4 determined by equation (1) and the proper isoclinal lines in the cross-section are shown in Fig. 3f and g, respectively.

In addition, to verify the existence of the twisted loop defect, we have performed the special structural and optical calculations (Fig. 3h,i), which illustrate obviously a good agreement of simulated texture with real image of considered droplet. The calculated director configuration in cross-section is perpendicular to the minor ellipsoid axis. The 3D twisted loop defect determined from the structure calculation is shown in Fig. 3h by red solid thick line in near half-space of the droplet and by the opaque red line in far half-space. As can see, the form of calculated twisted loop defect (Fig. 3h) is similar to the line disclination observed in experimental images. The simulated droplet texture in crossed polarisers (Fig. 3i) is in a good agreement with observed droplet pattern.

The orientations of bipolar axes within PDCLC film are random and, consequently, their optical textures can also be significantly different. In this case, understanding what orientational structure is formed in the droplet requires a detailed analysis of the optical texture by focusing on the identification of linear defect. For instance, Fig. 4 demonstrates the droplet with bipolar distribution of CLC axes and three isoclinal lines located symmetrically with respect to the droplet centre. The analysis of linear defect has shown that the orientation structure inside the droplet is similar to the one presented in Fig. 3. However, it is turned approximately by 90° around the bipolar axis aligning in the film plane (Fig. 4f,g).

The droplets of diameter *d* = 17 μm without isoclinal lines were found in the sample under study. However, by moving the microscope focus, the double left-handed helix with approximately 2.5 π turns is clearly seen (Fig. 5). Comparing the double helix with the linear defect shown in Figs 4 and 5, the droplet can be concluded to have a similar configuration, but the bipolar axis appears to be oriented at a small angle to the aspect direction (Fig. 5f). The analogous effect of isoclinal lines disappearance is observed within CLC droplets under a weak anchoring^18,22,23^. When the light propagates perpendicularly to the CLC axis, the lines periodically located at the distance of the half-pitch are clearly seen. And vice-versa, these lines disappear when observed along the CLC axis. The lesser *N* for the droplets of the same size evidences that LC droplets within the PDCLC film are oblate ellipsoidal^36,37^. Thus, the lateral droplet size is smaller than the one in the film plane.

**Figure 6:**
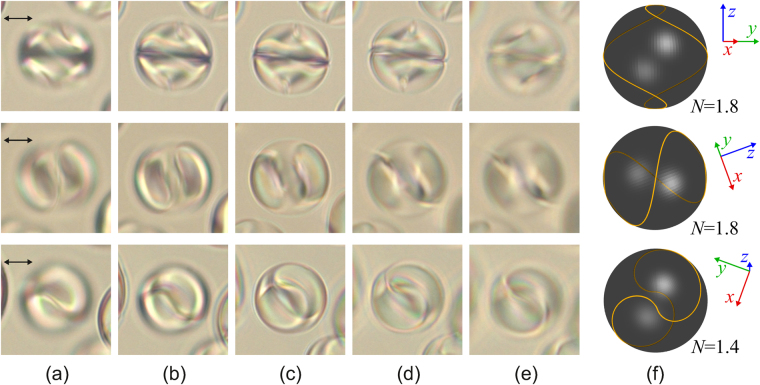
Micro-photos of CLC droplets made in the polarised light for various focusing of microscope. The focal point is located approximately at 1/6 (**a**), 2/6 (**b**), 3/6 (**c**), 4/6, (**d**), 5/6 (**e**) of the lateral size of droplet from the top border. The schemes of the proper linear defect *L* (**f**) observed along the *x*-axis (top row), along the *y*-axis (middle row) and when the bipolar axis (*z*-axis) is oriented at the 10° angle to the aspect direction (bottom row). The droplet size is 14 μm.

The orientation structure inside the droplets of about 14 μm is analogous to the ones described above with lesser *N*. The typical optical textures and the proper type of defect are shown in Fig. 6.

### Transformation of optical textures and twisted loop under electric field

**Figure 7:**
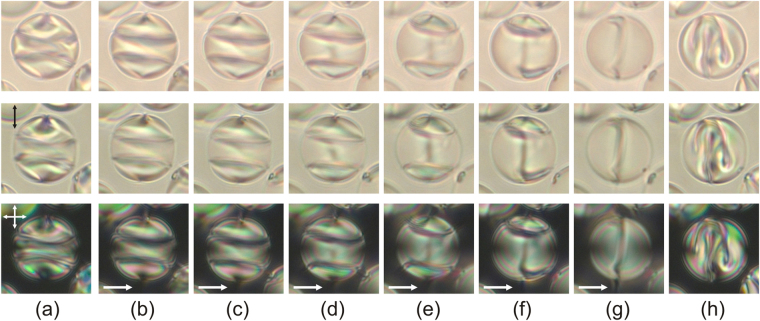
Micro-photos of the CLC droplet in the unpolarised light (top row), polarised light (middle row) and crossed polarisers (bottom row). The droplet textures in the initial state (**a**), at the voltages 112 V (**b**), 128 V (**c**), 149 V (**d**), 170 V (**e**), 192 V (**f**), 219 V (**g**), and immediately after the field breaking (**h**). The distance between the electrodes is 200 μm. The droplet size is 16 μm. The electric field direction is shown by a single arrow. The microscope focal point is approximately at the droplet centre.

Electric field affects the orientation structure of CLC droplets. Reorientation and the final CLC droplets configuration under an external field generally depend on the sign of dielectric anisotropy Δε, the initial orientation structure and the orientation of symmetry axis with respect to a field^25^. The nematic E7 has Δε > 0, consequently, the director tends to reorient along the electric field direction. Figure 7 demonstrates the response of the CLC droplet with two isoclinal lines to the electric field applied along the film plane perpendicularly to the bipolar axis. The response is nonthreshold and corresponds to a slight director reorientation at a low voltage. It changes the droplet interference colour observed in the crossed polarisers (Fig. 7a,b). The reorientation is accompanied by a gradual straightening of isoclinal lines. The further voltage increase results in the smooth rise of the distance between these lines. Here the isoclinal lines near the interface remain perpendicular to the border (Fig. 7c–e). Then, one of the isoclinal lines collapses into the droplet pole and disappears during several seconds under a small voltage increase (Fig. 7f). The continuing voltage increase makes the second isoclinal line collapse similarly (Fig. 7g).

**Figure 8:**
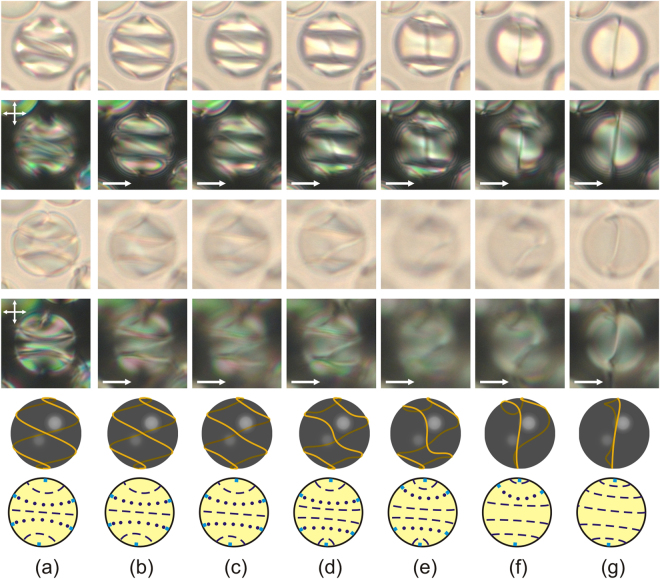
Micro-photos of the CLC droplet presented in Fig. 7 in the unpolarised light (first and third rows) and crossed polarisers (second and fourth rows). The microscope focal point is located approximately at 1/3 (first and second rows) and 2/3 (third and fourth rows) of the lateral size of droplet from the top border. The droplet textures in the initial state (**a**), at the voltages 112 V (**b**), 128 V (**c**), 149 V (**d**), 170 V (**e**), 192 V (**f**), 219 V (**g**). The electric field direction is shown by a single arrow. The fifth row presents the schemes of the double spiral defect and its transformation at applied voltage. Schematic description of corresponding director field in equatorial cross-section is shown in the sixth row.

The applied voltage makes the director in the bulk of CLC droplet reoriented and simultaneously changes the position and form of the linear defect. It is shown for the top and bottom parts of the same droplet (Fig. 8). As the voltage increases, the defect area between isoclinal lines tends to turn perpendicular to the applied field. The collapse of the isoclinal line leads to the decrease and following disappearance of a helix turn and appearance of a sharp bend of the defect line (Fig. 8g). As a result, under a sufficiently high voltage, the linear defect of double helix is transformed into the ring defect with two sharp bends whose the plane is oriented mainly orthogonally to the field. Finally, the untwisted axial-like structure with the linear defect oriented perpendicularly to the electric field is formed within the CLC droplet under the electric field (Figs 7g, 8g). The initial orientation structure is not restored just after electric field switching off, but a complex meta-stable structure appears (Fig. 7h). This meta-stable structure is restored into the initial one during two days. It should be noted the restoring time depends on many factors: temperature, voltage amplitude, droplet’s size, chirality parameter *N*, etc.

The droplet response at the initial state is generally similar to the response of the CLC bulk under a low electric field^38^, when the CLC axis tends to turn perpendicular to the field. Under the intermediate voltage, the areas of the CLC helix, where the director is oriented mainly parallel to the field, expand. Simultaneously, the areas where the director is mostly oriented perpendicularly to the field reduce. It causes the increase of the helix pitch and, eventually, the untwisting of the CLC structure into the achiral state under a high voltage. In correspondence with the experimental results described above, it can be concluded that the isoclinal lines observed within CLC droplets match those areas where the director is oriented preferably perpendicularly to the applied field. Since the director is oriented perpendicularly to the CLC axis, the isoclinal line corresponds to the droplet area where the director is oriented preferably perpendicularly to the film plane. This agrees with the above conclusion made on the position and form analysis of the linear defect within CLC droplets (Fig. 2).

## Conclusions

The orientation structures within the CLC droplets dispersed in polymer assigning the homeotropic anchoring have been experimentally studied and analysed. A number of CLC droplets reveal the optical textures where the lines are of circular arc shape and perpendicular to the visible droplet border. Such optical textures are characteristic to the structures with bipolar distribution of the CLC axes and they are formed inside droplets with homeotropic anchoring^18,25^. The sharp line corresponds to the area in the central droplet section where the director is mainly oriented perpendicular to the film plane. The bipolar configuration of the CLC axis is formed within droplets with *N* ≥ 2, that corresponds to *N*_0_ ≥ 5. Such a structure has the linear defect close to the surface in the form of the double left-handed helix similar to the twisted disclination loop predicted for *N*_0_ > 5 in simulation^26^. The light deviates due to the refractive index gradient in the droplet bulk and to the scattering effect at the linear defect. The latter is manifested in its optical texture. The brightest pattern of the defect scattering is seen when the bipolar axis is oriented close to the aspect direction. Here the isoclinal lines get invisible. Therefore, the shape and position analysis of the linear defect will allow determining the bipolar axis orientation within the CLC droplet. The droplets of equal size with various optical textures exemplify this, having the same orientation structure though (see Fig. 6).

To verify the existence of the twisted loop defect, the special structural and optical calculations have been performed. The simulated droplet texture in crossed polarisers is in a good agreement with observed droplet pattern. The form of calculated twisted loop defect is similar to the line disclination observed in experimental images.

The response of the CLC droplet to the field applied perpendicularly to the bipolar axis is generally similar to the response of the CLC bulk. Eventually, a structure with the ring linear defect is formed at high voltages. This structure is analogous to the axial configuration in nematics with the ring defect of +1/2 winding number^34,36,39^. The question of a defect type and its transformation during orientation structure changing is still of some interest. The CLC returns quickly into the twisting state after a sharp voltage switching-off. This process is partly similar to the case simulated in ref.^26^, where the CLC got quickly cooled from the isotropic phase. Consequently, the structures with a variety of bulk defects got formed and they further relaxed into a series of meta-stable states. In our experiment the droplet relaxes into another more complicated meta-stable structure after electric field switching-off. Here the topological defect should influence the relaxation scenarios of the orientation structure. The obtained results open up a possibility of the reversible switching of the droplet structure, which is promising for the development of memory effect materials.

## Methods

### Experimental approaches

The films of the polymer dispersed chiral-nematic liquid crystals (PDCLC) based on the poly(isobutyl methacrylate) (PiBMA) (Sigma) and the nematic E7 (Merck) doped with the left-handed chiral dopant cholesteryl acetate (Ch)^40^ have been studied. The weight ratio of the CLC components was E7: Ch = 97: 3. The intrinsic helix pitch of CLC *p*_0_ = 5.5 μm was measured by the droplet method^41^. The PDCLC films were made by the SIPS (solvent induced phase separation) method^36^. The glass substrate with two ITO electrode-strips placed at the 200 μm distance from each other was used to prepare the sample. The PDCLC 35 μm thickness films with CLC droplets of 5–30 μm size in the film plane were created. The optical textures of the chiral nematic droplets were examined by means of the polarising optical microscope Axio Imager.A1m (Carl Zeiss) with the 50x objective and 500x total magnification (resolution *R* ≅ 0.4 μm). The microscope was equipped by the camera AxioCam MRc5, allowing taking pictures with resolution 0.1 μm/pix for this magnification. The microscope permits us to take the pictures with 0.5 μm step along the light propagation (see Supplementary Figure 3). The design of electro-optical cells allowed us to observe the optical textures variation under the influence of ac electric field (1 kHz) applied along the film plane.

### Calculation of droplet structure

We performed calculations of LC structure within the oblate ellipsoidal cavity (axes ratio *x*:*y*:*z* = 1:1.4:1.4, rendered in a 48 × 48 × 34 lattice) filled with chiral nematic. We used the extended Frank elastic continuum approach with Monte-Carlo annealing optimization^42^ to find energy-optimal droplet structures. It previously shown good results for nematic and cholesteric LC^42,43^. This approach includes the effects of the director field distortion and the formation of defects in the droplet:

3 $$F={\int }_{V}[\frac{{K}_{11}}{2}{({\rm{div}}{\bf{n}})}^{2}+\frac{{K}_{22}}{2}{({\bf{n}}\cdot {\rm{rot}}{\bf{n}}+\frac{2\pi }{{p}_{0}})}^{2}+\frac{{K}_{33}}{2}{({\bf{n}}\times {\rm{rot}}{\bf{n}})}^{2}]dV+\frac{W}{2}{\int }_{\Omega }[1-{\cos }^{2}\vartheta ]d{\rm{\Omega }}+{F}_{def},$$

where *K*_11_, *K*_22_ and *K*_33_ are the splay, twist and bend elasticity constants, respectively, *W* is the surface anchoring energy density, *ϑ* is the angle between local director **n** and surface normal of the droplet, and *F*_def_ is the energy of defects calculated by the summation of the point and linear defect energies. The types, positions and energies of defects were estimated automatically during Monte-Carlo optimization procedure (see the details in ref.^42^). The ratio between elasticity constants was set to *K*_11_:*K*_22_:*K*_33_ = 1:0.6:1.25 to simulate the cholesteric liquid crystal mixture under study. Linear energy density of disclination core was set to *f*_core_^line^ = 10 *K*_11_, and the anchoring strength μ = *WR*/*K*_11_ = 400 was used for strong anchoring. R is the effective radius corresponding to the droplet of 17 μm size (Fig. 3). The equilibrium cholesteric pitch was set to *p*_0_ = 5.5 μm.

### Calculation of droplet picture in crossed polarisers

We calculated droplets textures using Jones matrices technique, formulated for PDLC materials in ref.^44^. This technique supposes direct unidirectional transition of linearly polarised light through non-uniform birefringent material. Light diffraction, diffusion and scattering are not taken into account in Jones calculus, thus textures on the peripheral parts of the droplets are roughly estimated. Textures were calculated for 10 wavelengths from visible spectrum, from 380 nm to 750 nm with equal step of 41 nm. Values of ordinary and extraordinary reflective indices of nematic E7 were set dependent on wavelength in accordance with^45^. Color textures were generated by merging individual wavelength textures with regards of experimental halogen lamp intensity at each wavelength.

## Electronic supplementary material

Supplementary Information
