# Supplementary material for: Bipolar configuration with twisted loop defect in chiral nematic droplets under homeotropic surface anchoring
Source: Sci Rep. 2017 Nov 6;7:14582. doi: 10.1038/s41598-017-15049-6 (PMC5674080; doi:10.1038/s41598-017-15049-6)

Supplementary Information

Bipolar configuration with twisted loop defect in chiral nematic droplets under homeotropic surface anchoring

Mikhail N. Krakhalev*[1,2], Anna P. Gardymova[2], Oxana O. Prishchepa[1,2], Vladimir Yu. Rudyak[3], Alexander V. Emelyanenko[3], Jui-Hsiang Liu[4], and Victor Ya. Zyryanov[1]

[1] *Kirensky Institute of Physics, Federal Research Center – Krasnoyarsk Scientific Center, Siberian Branch, Russian Academy of Sciences, Krasnoyarsk 660036, Russia*

[2] *Institute of Engineering Physics and Radio Electronics, Siberian Federal University, Krasnoyarsk 660041, Russia*

[3] *Faculty of Physics, Lomonosov Moscow State University, Moscow, 119991 Russia*

[4] *Department of Chemical Engineering, National Cheng Kung University, 70101 Tainan, Taiwan*

Corresponding author: M.N. Krakhalev, e-mail: kmn@iph.krasn.ru

Supplementary Figure 1-3

Supplementary Figure 1| POM photos of the CLC droplets with various structures were taken in the unpolarised light (top row), polarised light (middle row) and crossed polarisers (bottom row). Here and further, the polarisers directions are marked by double arrows. Droplet sizes are shown above the photos. Most structures (c-h) contain the linear defect of various form and they can be identified by POM. A number of the similar structures has been described earlier in: ref. [1, 2] (b), ref [3] (e), ref. [4] (g), ref. [5] (f).

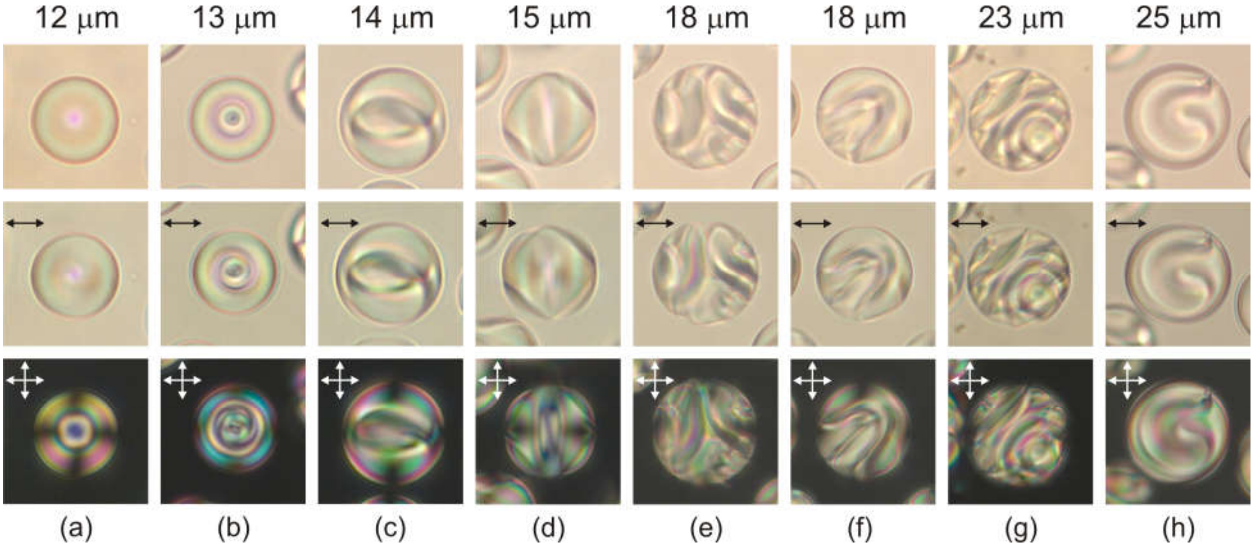

References

1. Posnjak, G., Copar, S. & Musevic, I. Points, skyrmions and torons in chiral nematic droplets. *Sci. Rep.* **6**, 26361; 10.1038/srep26361 (2016).
2. Orlova, T., Aβhoff, S. J., Yamaguchi, T., Katsonis, N. & Brasselet, E. Creation and manipulation of topological states in chiral nematic microspheres. *Nat. Commun.* **6**, 7603; 10.1038/ncomms8603 (2015).
3. Pierron, J. *et al*. Three-dimensional microstructure of a polymer-dispersed liquid crystal observed by transmission electron microscopy. *J. Phys. II* **5**, 1635-1647 (1995).
4. Bouligand, Y. & Livolant, F. The organization of cholesteric spherulites. *J. Phys.* **45**, 1899-1923 (1984).
5. Sec, D., Copar, S. & Zumer, S. Topological zoo of free-standing knots in confined chiral nematic fluids. *Nat. Commun.* **5**, 3057; 10.1038/ncomms4057 (2014).

Supplementary Figure 2| The double spiral (twisted loop) defect is the combination of two symmetrical spirals relatively to *z*-axis (c). Two diametrically opposite defect points are in each xy cross-section of the droplet. The first spiral (green curve, a) is realized when positive sign in equation for x and negative sign in equation for y. And vice versa, the second spiral (orange curve, b) is realized when negative sign in equation for x and positive sign in equation for y.

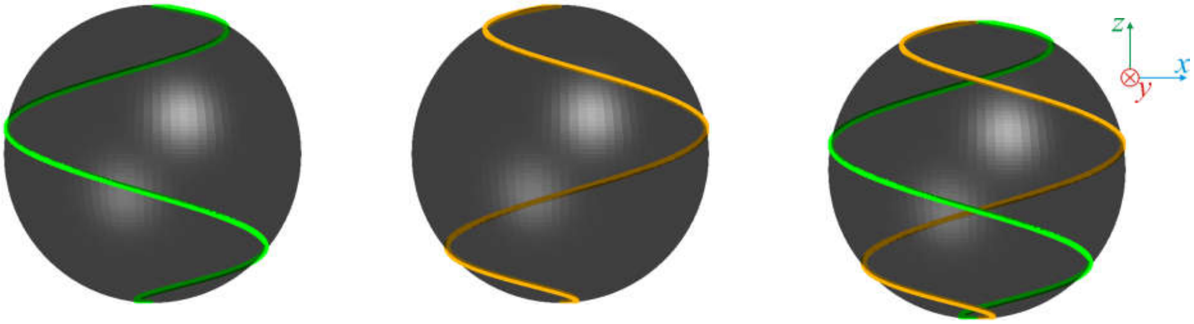

(a)

$$\begin{cases} x = +\sin\varphi \cdot \sin(N\varphi) \\ y = -\sin\varphi \cdot \cos(N\varphi) \\ z = \cos\varphi \\ 0 \le \varphi \le \pi \end{cases}$$

(b)

$$\begin{cases} x = -\sin\varphi \cdot \sin(N\varphi) \\ y = +\sin\varphi \cdot \cos(N\varphi) \\ z = \cos\varphi \\ 0 \le \varphi \le \pi \end{cases}$$

(c)

$$\begin{cases} x = (-1)^{k+1} \sin\varphi \cdot \sin(N\varphi) \\ y = (-1)^{k} \sin\varphi \cdot \cos(N\varphi) \\ z = \cos\varphi \\ 0 \le \varphi \le \pi, \quad k = 1,2 \end{cases}$$

Supplementary Figure 3| POM photos of the CLC droplet taken in the polarised light sequentially while the sample is vertically shifted relatively to the objective with a step Δh = 0.5 μm. As can see, the twisted defect loop is obviously identified using the POM method.

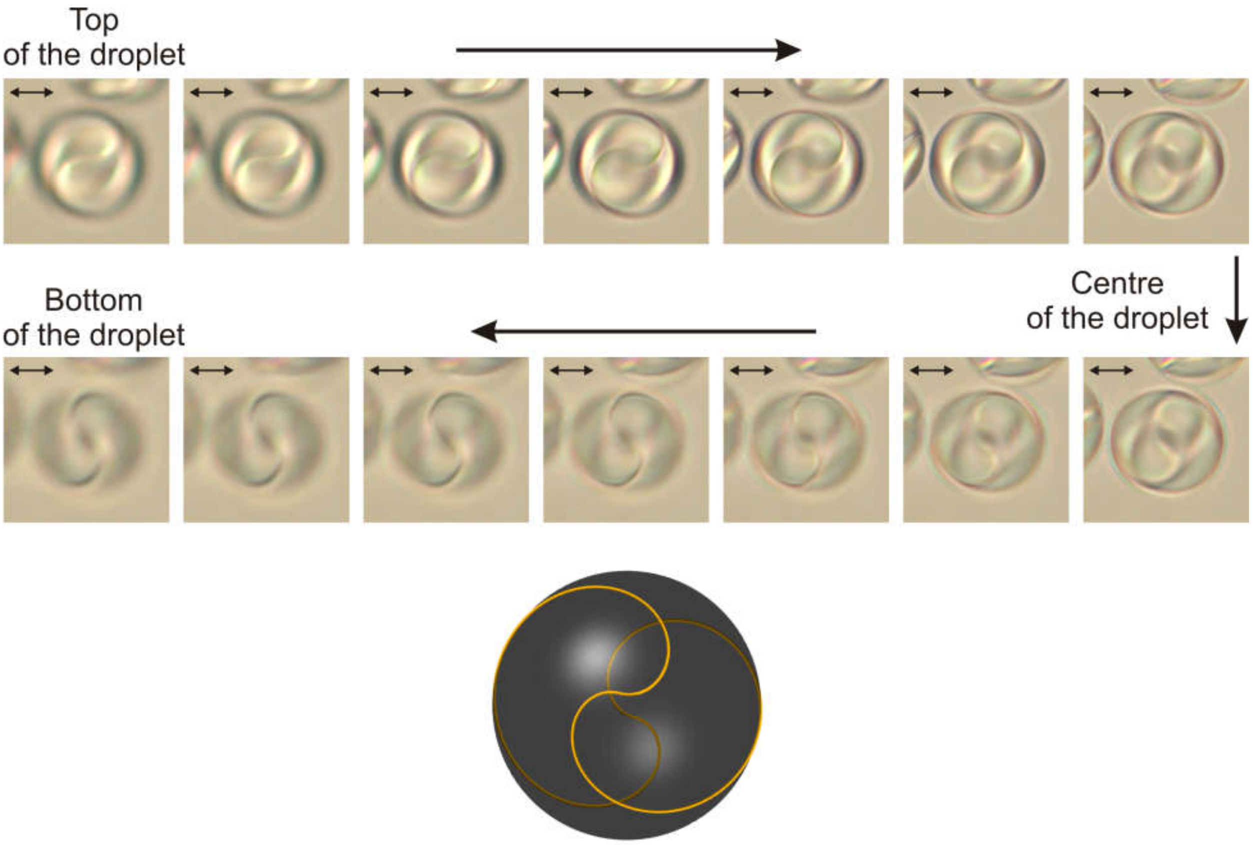

Supplement: Supplementary file 1 — Supplementary Information [file 41598_2017_15049_MOESM1_ESM.pdf]
